# Acute acalculous cholecystitis in a patient with dengue fever: A case report

**DOI:** 10.1016/j.amsu.2022.104960

**Published:** 2022-11-17

**Authors:** Shekhar Gurung, Saurab Karki, Manoj Khadka, Samanta Gurung, Sandesh Dhakal

**Affiliations:** a Chattarapati Free Clinic Community Hospital, Kathmandu, Nepal; b Military Hospital Itahari, Sunsari, Nepal; c Nepalese Army Institute of Health Science, Kathmandu, Nepal; d Aruchanute Primary Health Care Center, Gorkha, Nepal; e College of Medical Sciences, Chitwan, Nepal

**Keywords:** Acalculous cholecystitis, Case report, Dengue

## Abstract

**Introduction:**

and importance: Although dengue fever classically presents with fever, headache, retro-orbital pain, myalgia, arthralgia, and vomiting, it can have unusual manifestations like acalculous cholecystitis. The study highlights the importance of atypical presentations of dengue fever in suspecting dengue earlier, especially during outbreaks.

**Case presentation:**

Herein, we report a case of a 29 years old female who presented with fever for 5 days which was associated with headache, body ache, vomiting, and abdominal pain in the right hypochondriac region. Lab results came positive for dengue NS1 antigen, and ultrasonography showed features suggestive of acalculous cholecystitis. She was managed conservatively after which her symptoms resolved gradually.

**Clinical discussion:**

Acute acalculous cholecystitis in dengue could be due to increased vascular permeability leading to edematous thickening of the gall bladder wall. It should be suspected if a patient presents with fever, right upper quadrant pain, abnormal liver function tests, and thickened gall bladder wall without stones on abdominal ultrasonography.

**Conclusion:**

Acute acalculous cholecystitis is an atypical presentation of dengue fever. Awareness of atypical presentations of dengue helps in identifying dengue earlier and preventing complications.

## Highlights

• Dengue fever is caused by Dengue virus and transmitted by bite of infected *Aedes aegypti* and *Aedes albopictus* mosquitoes.
• Atypical manifestations include hepatitis, acalculous cholecystitis, pleural effusion, acute renal failure, encephalitis, myocarditis, and bleeding gastric ulcers.
• Ultrasonography is an important modality for early diagnosis whenever clinical picture of acalculous cholecystitis is confusing.
• Dengue is usually a self-limited condition and acute acalculous cholecystitis usually can be managed conservatively unless complicated by diffuse peritonitis.

## 1 Introduction

Dengue fever is caused by the Dengue virus that is transmitted by the bite of infected female *Aedes aegypti* and *Aedes albopictus* mosquitoes [1,2]. It classically presents with high fever, severe headache associated with retro-orbital pain, myalgia, arthralgia, vomiting, and rash [1,2]. Apart from dengue fever, dengue infection can manifest as dengue hemorrhagic fever (DHF), dengue shock syndrome (DSS), and expanded dengue syndrome (EDS) which includes the atypical manifestations of dengue fever [3]. Some unusual manifestations of dengue fever include hepatitis, acalculous cholecystitis [[3], [4], [5], [6], [7], [8], [9], [10]], pleural effusion, acute renal failure, encephalitis, myocarditis, and bleeding gastric ulcers [3,11].

Herein we report a case of atypical presentation of dengue fever in a 29 years old female in the form of acute acalculous cholecystitis. The study highlights the importance of unusual manifestations of dengue fever which can help clinicians identify dengue earlier, especially during outbreaks. This case has been reported in line with the SCARE 2020 criteria [12].

## 2 Case presentation

29 years old normally menstruating female, non-smoker and non-alcoholic presented with fever for 5 days and abdominal pain for 1 day. Fever was continuous with a maximum temperature recorded of 102° Fahrenheit, break-bone type, associated with chills and rigors, and partially controlled by taking medications. There was a history of headache, body ache, decreased appetite, and multiple episodes of non-bilious, non-projectile, and non-blood-stained vomiting. She also had pain for a day in the right hypochondriac region, sharp in nature, non-radiating aggravated by taking deep inspiration, and relieved by taking analgesics. There is no history of loss of consciousness, bleeding from any orifices, rashes over the body, shortness of breath, or cough. Her bowel and bladder habits were normal.

On examination, she was looking ill, averagely built, and well oriented to time, place, and person. At presentation, her temperature was 100° Fahrenheit with normal blood pressure, pulse rate, and oxygen saturation. Icterus was noted over bilateral bulbar conjunctiva with no other significant findings on general examination. There was tenderness noted over the right hypochondriac region, however, there was no guarding or rigidity. No organs could be palpated and her bowel sounds were heard. Murphy's sign could not be elicited. Examination of respiratory, cardiovascular, and neurosensory systems was non-revealing.

**Fig. 1:**
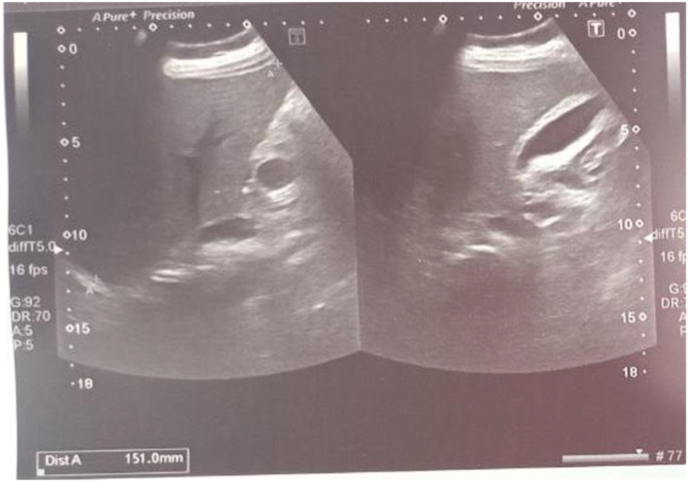
Ultrasonography of abdomen showing normal sized gall bladder with increased wall thickness (5.7mm) suggestive of acute acalculous cholecystitis.

On investigations, her hemoglobin level was 12.8 g/dl, hematocrit was 36.5% with total leukocyte counts of 2100 cells/cubic millimeter and platelet count of 108,000 cells/cubic millimeter. Liver function tests revealed raised bilirubin levels (total serum bilirubin of 6.5 mg/dl and direct serum bilirubin level of 3.5mg/dl) with raised liver enzymes (ALT 224U/L, AST 412U/L, ALP 710U/L). Renal function tests were within normal limits. As there was a dengue outbreak in the city, her NS1 was positive for dengue but serology was non-reactive. Rapid Diagnostic Test (RDT) for COVID-19 and malaria was negative. Serology for hepatitis B, C, and ELISA for HIV was non-reactive. Ultrasonography of the abdomen showed normal sized gall bladder with increased wall thickness (5.7mm) and no space-occupying lesion, features suggestive of acalculous cholecystitis as shown in Fig. 1. Further, there was mild splenomegaly with spleen size measuring 14.2 cm with normal outline and echotexture but no focal lesions. Based on clinical, pathological, and radiological parameters, a diagnosis of dengue fever with acute acalculous cholecystitis was made.

With this diagnosis, the patient was admitted and managed conservatively with intravenous fluids, paracetamol and ceftriaxone to prevent secondary bacterial infections. She was advised for soft diet and multivitamins were added to improve liver function. Her daily complete blood count was monitored. With the resolution of fever and dengue symptoms, icterus also decreased, right hypochondriac pain subsided and liver functions started to reach baseline. She will be followed up with an USG to look for resolution of acalculous cholecystitis in 1 month.

## 3 Discussion

Dengue is a mosquito-borne viral infection caused by an RNA virus of the family Flaviviridae, dengue virus. Earlier patients were classified as having dengue fever, dengue hemorrhagic fever, or dengue shock syndrome, however, World Health Organization (WHO) has recently classified patients as having either dengue with or without warning signs or severe dengue [1,2]. Clinical manifestations of dengue follow three phases, febrile, critical, and recovery phase [2]. It classically presents with high fever, headache, vomiting, myalgia, arthralgia, and often a macular rash [2]. However, it can have unusual manifestations too.

Expanded dengue syndrome is a terminology added by the WHO to include atypical manifestations of dengue involving the liver, gut, kidney, heart, or brain [3,11]. Awareness of the unusual presentations of dengue helps in identifying dengue early, especially during outbreaks [3]. Some atypical neurologic manifestations are encephalopathy, encephalitis, and, intracranial hemorrhages, cardiac manifestations being myocarditis, and pericarditis, hepatic presentation as hepatitis, acute liver failure, and acalculous cholecystitis, and renal presentation as acute renal failure [3]. Endothelial dysfunction, increased vascular permeability, and coagulation disorders are the key mechanisms for the involvement of these various organ systems in dengue [2,11].

Acute acalculous cholecystitis is the inflammation of the gallbladder in absence of calculi. It should be suspected if a patient presents with fever, right upper quadrant pain, abnormal liver function tests, and thickened gall bladder wall without stones on abdominal ultrasonography [10]. Our patient also had these features. Acute acalculous cholecystitis in dengue could be attributed to increased vascular permeability leading to edematous thickening of the gallbladder wall [3,10]. Since dengue is a self-limited viral infection, the thickening of the gallbladder wall could be reversible, suggesting surgical management of acute acalculous cholecystitis in dengue patients mightn't be necessary initially unless complicated by diffuse peritonitis [10]. In a study conducted in a Taiwan hospital during a dengue outbreak, 10 out of 131 dengue patients had acute acalculous cholecystitis, among which three underwent surgical treatment and had complications like bleeding and shock delaying the discharge than those not undergoing invasive treatment [10].

Acute acalculous cholecystitis can progress to gangrene, empyema, or perforation, so early identification is crucial [13]. Ultrasonography is an important modality for early diagnosis whenever the clinical picture is confusing [13]. The findings in acalculous cholecystitis are the absence of stones, thickened gallbladder wall, enlarged tender gallbladder, and pericholecystic fluid collection [13]. Our case had thickened gallbladder wall with an absence of stones within the gallbladder.

## 4 Conclusion

Acute acalculous cholecystitis in a dengue patient should be suspected if they present with fever, right upper quadrant pain, abnormal liver function tests, and thickened gall bladder wall without stones on abdominal ultrasonography. Clinicians need to be aware of the unusual manifestations of dengue to identify it earlier, especially during dengue outbreaks for better clinical outcomes and to prevent complications.

## Authors contributions

Author 1: Led data collection, concept of study, literature review.

Author 2: Literature review, revising, and editing the rough draft into final manuscript.

Author 3: Literature review, writing manuscript draft, revising, and editing the manuscript.

Author 4: Literature review, revising and editing the manuscript.

Author 5: Literature review, revising and editing the manuscript.

All authors were involved in manuscript drafting and revising, and approved the final version.

## Sources of funding

None.

## Ethical approval

N/A.

## Consent

Written informed consent was obtained from the patient for publication of this case report and accompanying images. A copy of the written consent is available for the review by the Editor-In-Chief of this journal on request.

## Research registration

N/A.

## Guarantor

Dr. Saurab Karki, Militiary Hospital, Itahari-4, Sunsari, Nepal. Email: saurabkarki1010@gmail.com, Phone: +977–9841098336.

## Provenance and peer review

Not commissioned, externally peer-reviewed.

## Registration of research studies

1. Name of the registry: N/A.
2. Unique Identifying number or registration ID: N/A.
3. Hyperlink to your specific registration (must be publicly accessible and will be checked): N/A.

## Declaration of competing interest

None.
